# Multiple transcatheter interventions in the same session in congenital cardiopathies

**DOI:** 10.4103/0975-3583.74261

**Published:** 2010

**Authors:** Saritas Turkay, Erdem Abdullah, Akdeniz Celal, Zeybek Cenap, Erol Nurdan, Demir Fadli, Demir Halil, Aydemir Numan Ali, Celebi Ahmet

**Affiliations:** *Dr. Siyami Ersek Thoracic and Cardiovascular Surgery Center and Research Hospital, Istanbul, Turkey*; 1 *Dr. Siyami Ersek Thoracic and Cardiovascular Surgery Center and Research Hospital, Pediatric Cardiology, Istanbul, Turkey*; 2 *Dr. Siyami Ersek Thoracic and Cardiovascular Surgery Center and Research Hospital, Cardiovascular Surgery, Istanbul, Turkey*

**Keywords:** Congenital cardiac defects, percutaneous procedures, percutaneous treatment

## Abstract

**Background:**

To investigate the methods of percutaneous transcatheter interventions for combined congenital heart disease and to evaluate its efficacy in children.

**Materials and Methods:**

Thirty cases (ages 3 days-13.5 years, body weight 3-35 kg) that underwent two multiple transcatheter interventions for combined or solitary congenital heart disease were retrospectively analyzed and presented.

**Results:**

A total of 61 transcatheter interventions were performed in 30 patients as combined procedures. In 29 patients, two different procedures were combined in the same session, in remaining one patient, combination of three interventions were performed in the same catheter session. Interventions performed in combined procedures were as follows: Coarctation balloon angioplasty in 12 cases, pulmonary balloon valvuloplasty in 10, PDA coil embolization in 10, aortic balloon valvuloplasty in 8, VSD closure in 5, ASD closure in 4, ductal stent implantation in 4, palliative pulmonary balloon valvuloplasty in 3, recanalization and angioplasty of the systemic-pulmonary shunt in 2, balloon atrial septostomy in one, aortic coarctation stent implantation in one, coil embolization of a pulmonary lobar sequestration in one and pulmonary valve perforation plus pulmonary balloon valvuloplasty in one were performed as first or second procedure. There was no mortality or major morbidity in relation to combined procedures.

**Conclusion:**

Multiple transcatheter interventions in the same session are feasible, safe and effective with satisfactory good results. Second intervention may be performed as complementary procedure or independently to the first intervention.

## INTRODUCTION

Advances in pediatric interventional cardiac catheterization have changed the therapeutic strategy for many patients with congenital heart disease. Since the first attempt of palliation of transposition of the great arteries by transcatheter technique,[1] the efficiency and safety of transcatheter interventional treatment have been greatly improved. Nowadays, many congenital cardiac diseases can be treated by transcatheter interventional procedures.[2–4] Moreover for some congenital lesions, such as pulmonary valvular stenosis,[5] transcatheter therapy nearly replaced the surgery.

For combined congenital cardiovascular defects, the efficacy and safety of transcatheter interventional therapy has been a topic of increasing concern. By the late nineties, reports of an interventional procedure prior to surgery for combined defects emerged, but there are only a few case reports where multiple defects were addressed simultaneously by interventional procedures.[1]

We present our experience of percutaneous transcatheter interventions for combined congenital heart diseases and discuss the efficacy and safety of these simultaneous procedures in children.

## MATERIALS AND METHODS

From the years 2004-2010, a total of 30 children (15 boys, 15 girls) underwent combined transcatheter interventions for multiple congenital heart defects. After routine physical and radiological evaluation, transthoracic echocardiographic examination was performed to all patients using Vivid-3 (General Electric) machine with 3.5 and 5 MHz transducers. Diagnostic cardiac catheterization and all interventional procedures were performed under general anesthesia.

A total of 61 transcatheter interventions were performed in 30 patients as combined procedures. In 29 patients two different procedures were combined in the same session; in remaining one patient combination of three interventions were performed in the same catheter session. Interventions performed in combined procedures were as follows:

Balloon angioplasty for coarctation to 12 children, pulmonary balloon valvuloplasty to 12 patients, transcatheteter coil embolization to 10 children, aortic balloon valvuloplasty to eight patients, ventricular septal defect (VSD) closure to five patients, atrial septal defect (ASD) closure to four patients, ductal stent implantation to four patients, recanalization of occluded Blalock-Taussiq shunt in two children, perforation of the pulmonary valve in one child, coil embolization of pulmonary sequestration in one child, balloon atrial septostomy to one child, and stent implantation for coarctation of the aorta in one child.

For valvular stenosis, peak systolic pressure gradients and for aortic coarctation, peak-to-peak systolic pressure gradients were determined before and after valve dilatation and/or aortic coarctation angioplasty and stenting.

Because the whole defects are small, non-detachable Gianturco coils were used for coil embolization of the patent ductus arteriosus. According to the nature of the defect and to the accompanying disease coil implantation were performed from arterial or venous sides of the ductus arteriosus.

Defect occlusion for ASDs and VSDs were performed under transesophageal or transthoracic echocardiographic guidance. All children with implanted devices received intravenous heparin (100 IU/kg) and prophylactic antibiotics during the procedure. Antithrombotic dose of acetylsalicylic acid was prescribed to all children with device closure for 6 months.

Ductal stent implantation was performed transarterially with standard coronary type stent in patients with pulmonary atresia to access effective flow to the pulmonary tree. After implantation of stent to ductal location and being sure of effective pulmonary flow, retrograde pulmonary valve perforation with a guide-wire and then balloon valvuloplasty to perforated valve were performed in selected cases. Intravenous heparin and acetylsalicylic acid were used sequentially after stent implantation procedure.

### Statistical evaluation

Differences among pre- and post-procedure in cases who performed balloon angioplasty for coarctation of aorta, aortic balloon valvuloplasty and pulmonary balloon valvuloplasty were analyzed separately with the Student’s *t* test. *P*-values below 0.05 were considered statistically significant.

## RESULTS

### Study population

The mean age of our whole study population was 36.3 ± 48.1 months (4 days–13.5 years) and the mean body weight was 12.4 ± 9.8 kg (2.2-35 kg). Age, weight and diagnosis of the whole cases is shown in Table 1.

**Table 1:** Age, weight and diagnosis of the whole cases

Cases		Sex	Age	Weight (Kg)	Diagnosis
1	ANB	F	1 month	3	AoCo, Bicuspid AV, AS, LVH, PFO
2	SY	M	6 months	7.5	Bicuspid AV, AS, AoCo
3	EE	M	11.5 years	35	AoCo, PS
4	MK	M	10 years	28.5	AoCo, Pm VSD, AI (mild)
5	SNC	F	42 days	3.5	Severe AS, discrete AoCo, PFO, LVH, severe PH
6	MM	M	5 months	6.8	AoCo, bicuspid AV, AS, LVH, trivial Mitral insufficiency
7	SEB	M	7 days	3.8	AoCo, bicuspid AV, AS, LVH, PDA, PFO, severe PH
8	SP	F	2 months	3,5	Dextro-transposition of great arteries, VSD, AoCo, PFO, PDA
9	AB	M	15 months	8	AoCo, PDA, bicuspid AV, AS (mild)
10	NA	F	17 months	10	Operated VSD+ASD+PDA, residual PDA, AoCo (mild)
11	SC	F	4 years	16	PS, PDA
12	AA	M	13.5 years	35	Pm VSD, VSA, AV prolapse, PDA
13	RB	M	5.5 years	22	Pm VSD, VSA, PDA
14	HG	F	6.5 years	25.5	Secundum ASD, PDA, bicuspid AV, trivial AI
15	HT	M	3.5 months	8.5	Bicuspid AV, AS, PS, Biventricular hypertrophy, PFO
16	NAO	M	20 days	3.8	Shone complex, critic AoCo, Mitral stenosis (mild), bicuspid AV, AS, Pm VSD, PDA, left ventricular hypoplasia (mild)
17	EK	F	2.5 years	8.5	TOF, right arcus aorta, right Modified BT shunt (Obstructed)
18	KA	F	4 days	3.4	Critic PS, PFO, PDA
19	BNK	F	24 days	2.2	Hypoplastic tricuspid valve, severe tricuspid stenosis, hypoplastic bipartiete right ventricle, critic PS, small Muscular VSD, AI, large secundum ASD, PDA
20	ST	F	14.5 months	9.5	Pulmonary atresia with intact ventricular septum, PDA
21	YEC	M	1 month	3.2	AoCo, Pm VSD, left ventricle-right atrium communication, left ventricular systolic dysfunction, PFO, pulmonary sequestration
22	ENV	F	23 months	11	PDA (mild size), secundum ASD
23	MS	M	8,5 years	24	Semidysplastic pulmonary valve, severe PS, secundum ASD, right ventricular hypertrophy
24	FC	F	8.5 years	19	Bicuspid AV, severe AS, long segment AoCo, LVH
25	ZNT	F	13 months	10,6	PS, PDA (Small-Moderate)
26	BM	M	13 months	8	Pm VSD (Moderate), VSA, PDA
27	EK	F	6 months	6	Double inlet left ventricle, double outlet left ventricle, PS, Left Modified BT shunt (Obstructed)
28	YB	M	3 years	12,3	Down Syndrome, TOF, Left Modified BT shunt (Obstructed)
29	KK	F	10.2 years	23	PS, Secundum ASD
30	BE	M	11 years	44	Pm VSD, VSA, AV prolapse, AI (mild), PDA (small)

F: Female, M: Male, AoCo: Aortic coarctation, AI: Aortic insufficiency, AS: Aortic stenosis, ASD: Atrial septal defect, AV: Aortic valve, LVH: Left ventricular hypertrophy, PDA: Patent ductus arteriosus, PFO: Patent foramen ovale, PH: Pulmonary hypertension, PS: Pulmonary stenosis, Pm: Perimembranous, TOF: Tetralogy of Fallot, VSD: Ventricular septal defect, VSA: Ventricular septal aneurysm.

### Procedural features

Coarctation balloon angioplasty was the most performed procedure in our study population, performed in 12 cases. Coarctation balloon angioplasty was performed as the former procedure in four cases, as the latter procedure in eight cases. Lesions were native in all cases [Table 2]. The mean age of these cases was 25.5 ± 48.9 months (2 days-11.5 years) and mean weight was 9.7±10.6 kg’s (3-35 kg). The mean gradient across the lesion was 23.2 ± 13.2 mmHg (1-44 mmHg) before and 7.7 ± 4.2 mmHg (0-15 mmHg) after balloon angioplasty (*P*=0.001).

**Table 2:** First and second procedures and their outcomes

Cases	First procedure	Second procedure	Outcomes
1	ABV		Transaortic gradient decreased from 56 to 23 mmHg.
			AoCo gradient decreased from 44 to 12 mmHg.
2	ABV		Transaortic gradient decreased from 64 to 30 mmHg.
			Aortic coarctation gradient decreased from 12 to 7 mmHg.
3	PBV		Total transpulmonic gradient decreased from 46 to 8 mmHg.
			AoCo gradient decreased from 18 to 9 mmHg.
4	VSD closure using coil	BAP	VSD was closured totally. AI was reduced from mild to trivial.
			AoCo gradient decreased from 30 to 10 mmHg.
5	ABV		Transaortic gradient decreased from 45 to 20 mmHg. Mild AI was developed.
			AoCo gradient decreased from 15 to 5 mmHg.
6	ABV		Transaortic gradient decreased from 40 to 20 mmHg.
			AoCo gradient decreased from 32 to 11 mmHg.
7	ABV		Transaortic gradient decreased from 30 to 25 mmHg.
			AoCo gradient decreased from 7 to 5 mmHg.
8	BAS		SaO2 increased from 68 to 84%, and interatrial gradient decreased from 6 to 2 mmHg after BAS. AoCo gradient decreased from 22 to 15 mmHg.
9	BAP		AoCo gradient decreased from 30 to 4 mmHg. PDA was closed totally.
10	BAP		AoCo gradient decreased from 42 to 11 mmHg. PDA was closed totally.
11	PBV		Total transpulmonic gradient decreased from 60 to 25 mmHg.
			Minimal residual shunt was observed from PDA.
12	VSD closure using ADO	Coil embolization of PDA	Both defects were closed totally. Complet atrioventricular block was developed.
13	VSD closure using ADO		Both defects were closed totally.
14	ASD closure using ASO		Both defects were closed totally.
15	PBV	ABV	Transaortic gradient decreased from 70 to 10 mmHg. Mild AI was developed.
			Total transpulmonic gradient decreased from 87 to 30 mmHg.
16	BAP		In this case who has systolic dysfunction, indentation lost during both procedures.
			However, at the beginning and end of both procedures, there was no gradient in both lesions due to systolic dysfunction.
17	Modified BT shunt recanalisation	Palliative PBV	SaO2 increased from 60 to 75% after recanalization of BT shunt.
			SaO2 increased to 81% after second procedure.
18	PBV	Ductal stenting	Despite total transpulmonic gradient decreased to 25 mmHg the compliance of right ventricle and saturation didn’t improve, and ductal stenting was performed.
			SaO2 increased from 62 to 92% after ductal stenting.
19			SaO2 increased from 70 to 80 % and total transpulmonic gradient decreased to 20 mmHg after first procedure. SaO2 increased from 80 to 90 % after second one.
20	Ductal stenting	Perforation of the PV and than PBV	SaO2 increased from 40 to 65% after ductal stent implantation.
			SaO2 increased to 75%-80% after third procedure.
21	BAP	Coil embolization of the pulmonary sequestration	AoCo gradient decreased from 42 to 11 mmHg.
			Pulmonary sequestration was embolizated successfully.
22	Coil embolization of PDA	ASD closure using CSO	Both defects were closed totally.
23	PBV		Transpulmonary gradient decreased from 128 to 7 mmHg.
			ASD was closed totally.
24	ABV	Stent implantation for AoCo	Transaortic gradient decreased from 118 to 45 mmHg. Moderate AI was developed.
			AoCo gradient decreased from 36 to 0 mmHg.
25	PBV	Coil embolization of PDA	Total transpulmonic gradient decreased from 78 to 11 mmHg.
			PDA was closed totally.
26	VSD closure using ADO II	Coil embolization of PDA	Minimal residual shunt was observed from VSD.
			PDA was closed totally.
27	Palliative PBV	Ductal stenting	SaO2 increased to 72% after first procedure, and increased to 78 after second one.
28	Modified BT shunt recanalisation	Palliative PBV	SaO2 increased from 63 to 74% after first procedure, and increased to 96 after second one.
29	ASD closure using CSO	PBV	ASD was closed totally. Transpulmonary gradient decreased from 53 to 15 mmHg.
30	VSA closure using muscular VSD occluder	Coil embolization of PDA	Both defects were closed totally.
			AI was reduced from mild to trivial.

ABV: Aortic balloon valvuloplasty, BAP: Balloon angioplasty of aortic coarctation, PBV: Pulmonary balloon valvuloplasty, VSD: Ventricular septal defect, BAS: Balloon atrial septostomy, PDA: patent ductus arteriosus, SaO2: Saturation, ADO: Amplatzer duct occluder, ASO: Amplatzer septal occluder, PV: Pulmonary valve, CSO: Cardi-O-Fix atriyal septal occluder, BT:Blalock-Taussig.

Pulmonary balloon valvuloplasty was performed as the former procedure in eight cases [Table 2]. The mean total transcatheter gradient across the pulmonary valve in the cases except in one who was performed palliative pulmonary valvuloplasty was 75.4 ± 28.7 mmHg (44-131 mmHg) before valvuloplasty and 14.1 ± 8.3 mmHg (7-30 mmHg) after valvuloplasty in these patients (*P*=0.001).

In a 24-days old baby weighing 2.2, with the diagnosis of hypoplastic tricuspid valve, severe tricuspid stenosis, hypoplastic right ventricle and critical pulmonary stenosis; pulmonary balloon valvuloplasty was performed with a 4-mm coronary balloon first, and then the procedure was repeated with a 7-mm standard balloon. The transpulmonic gradient was decreased, but oxygen saturation was between 70 and 80% after valvuloplasty. Ductal implantation of a 3.5 mm coronary stent was performed as a second procedure to achieve satisfactory oxygenation by an extra increase in pulmonary blood flow. The oxygen saturation at last was 90 % [Table 2].

In another 4-days old baby, with critical pulmonary stenosis, despite a decrease of total transpulmonic gradient to 25 mmHg after pulmonary balloon valvuloplasty, the compliance of right ventricle and systemic saturation did not improve. To overcome this situation, a 3.5 mm coronary stent was implanted retrogradely into ductus and systemic oxygen saturation increased from 62 to 92% [Table 2].

Pulmonary balloon valvuloplasty was performed as the second procedure in three cases (pulmonary valvuloplasty for antegrad palliation in two cases), and as the third procedure in one [Table 2]. In two patients with Fallot’s tetralogy, recanalization of previously constructed and obstructed surgical aortopulmonary shunt and then dilatation with a 4-mm coronary balloon was performed, but this much dilatation of the shunt could not be sufficient enough to increase patient’s oxygenation. Palliative pulmonary balloon valvuloplasty was performed as a second procedure with a sufficient increase in patient’s oxygenation [Table 2]. In another case with pulmonary atresia with intact ventricular septum, a 4-mm coronary stent was implanted firstly, but the oxygen saturation did not increase efficiently. For this reason, firstly pulmonary valve was perforated using guidewire through ductal stent, and then pulmonary balloon valvuloplasty was performed as third procedure. After the last procedure, the oxygen saturation increased up to 75-80% (Table 2, Movie 1 and 2).

**Figure 1:**
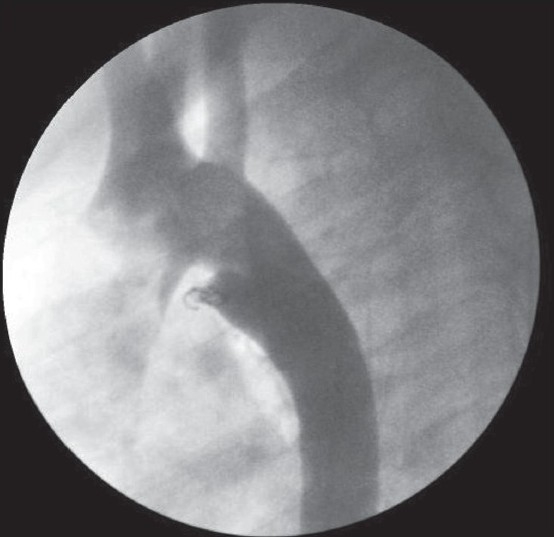
Coil embolization of PDA using Gianturco non-detachable coil as first or second procedure

PDA’s were embolizated by using Gianturco non-detachable coils in 10 cases. The mean size of the PDA on aortogram was 1.9±0.3 mm (1.5–2.5 mm). The mean diameter of the coils used in PDA closure was 4.2 ± 0.9 mm (3-5 mm). The stenosis in left pulmonary artery due to coil embolization was not detected in any of the patients. Full occlusion was seen in 9 patients, while a minimal residual shunt was detected in one patient [Figure 1, Table 2].

The mean age of the patients that were carried out aortic balloon valvuloplasty was 16.9 ± 3.8 months (4 days-103 months) and the mean weight was 7.4 ± 5.4 kg (3-19 kg). The mean annulus diameter was 9.5 ± 3.1 mm (6.3-14.5 mm), maximum systolic gradient was 71.7 ± 31.4 mmHg (19-115 mmHg) and mean systolic gradient was 45 ± 11.8 mmHg (32-62 mmHg) in transthoracic echocardiography. Aortic annulus diameter measured at catheterization laboratory was 8.4 ± 3.5 mm (4.5-14.4 mm). The mean diameter of balloon was 8.2 ± 3.1 mm (5-14 mm). Peak-to-peak transaortic gradient before procedure was 54.7 ± 35.6 mmHg (5-118 mmHg) at cath-lab. Peak-to-peak gradient was 20.5 ± 12.1 mmHg (0-36 mmHg) after balloon valvuloplasty (*P*=0.001). Mild aortic regurgitation in two cases and moderate aortic regurgitation in one were detected after valvuloplasty procedure [Table 2].

**Figure 2:**
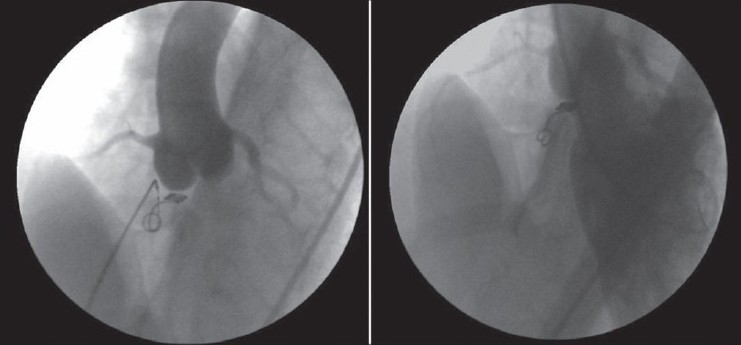
Closing of VSD using Cook’s detectable coil (5 mm’of diameter with 5 loop) and left ventriculogram shortly after implantation of coil

**Figure 3:**
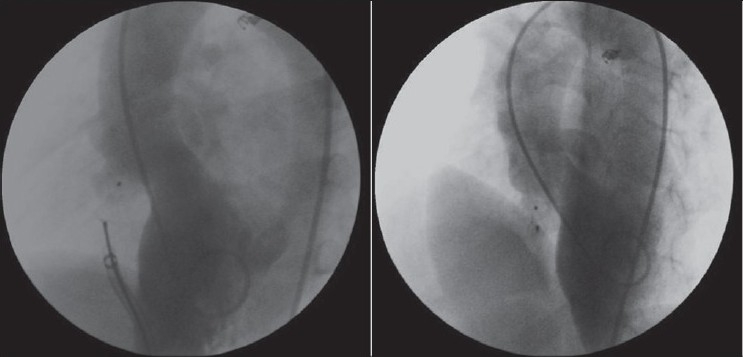
Closing of VSD using Amplatzer duct occluder 1 and left ventriculogram shortly after implantation of device

**Figure 4:**
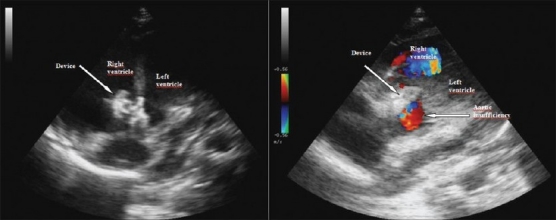
Closing of VSD using Amplatzer duct occluder 2 and checking of the aortic insufficiency shortly after releasing the device on transthoracic echocardiographic imaging

VSDs were closed by using coil in one, Amplatzer duct occluder 1 in two patients, Amplatzer duct occluder 2 in one patient and Cardi-O-Fix muscular VSD occluder in one patient [Table 2, Figure 2–5a–b]. Pulmonary arterial pressures were normal in all patients, but there were left heart dilatation. There was mild aortic insufficiency before the procedure in one case. In other case, too, there was ventricular septal aneursym, aortic valve prolapse and mild aortic insufficiency. The ratio of pulmonary to systemic flow (Qp/Qs) was not more than 1, 5 in these two cases. In the case who has only mild aortic insufficiency, the defect was closed by using Cook’s detectable coil (5 mm of diameter with 5 loop) with the guidance of transthoracic echocardiography. In the case of a patient who has ventricular septal aneursym, aortic valve prolapse and mild aortic insufficiency, the defect in ventricular septal aneursym in stead of true defect was closed by using 6 millimeter Cardi-O-Fix muscular VSD occluder with the guidance of transthoracic echocardiography. The aortic regurgitation decreased from mild to trivial amount after procedure in both cases.

**Figure 5a:**
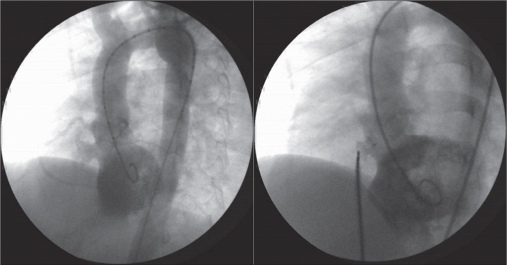
Closing of VSD using Cardi-O-Fix muscular VSD occluder and checking of residual shunt shortly after releasing the device on left ventriculogram in the long-axial oblique projection

**Figure 5b:**
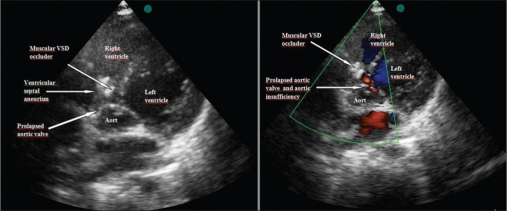
Closing of VSD using Cardi-O-Fix muscular VSD occluder and checking of the aortic insufficiency shortly after releasing the device on transthoracic echocardiographic imaging

Qp/Qs was more than 1, 5 in the other three cases. They had ventricular septal aneurysms. These defects were closed by using Amplatzer duct occluder 1 and 2 with the guidance of transthoracic echocardiography. In one of them, a complete atrioventricular block was developed in a short time after device implantation. There was no residual shunt on left ventriculogram in the long axial oblique projection except one patient. Minimal residual shunt was observed in case who was used Amplatzer duct occluder 2 after procedure [Figures 4–5b].

There observed no tricuspid regurgitation related to VSD closure in any of the patients. We did not detect any increase in the severity of aortic regurgitation related to the procedure in any patient.

**Figure 6:**
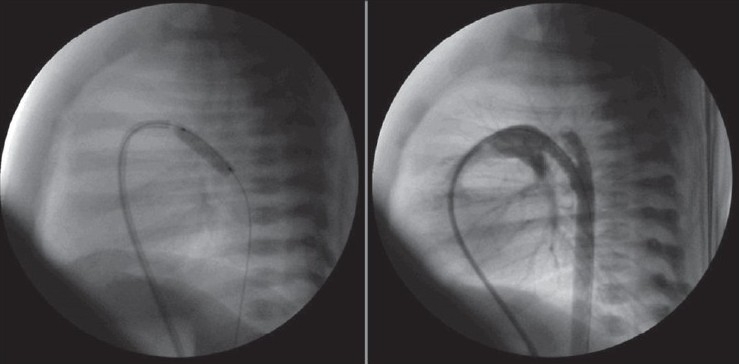
Ductal stenting after pulmonary balloon valvuloplasty in a case with critic pulmonary stenosis

**Figure 7:**
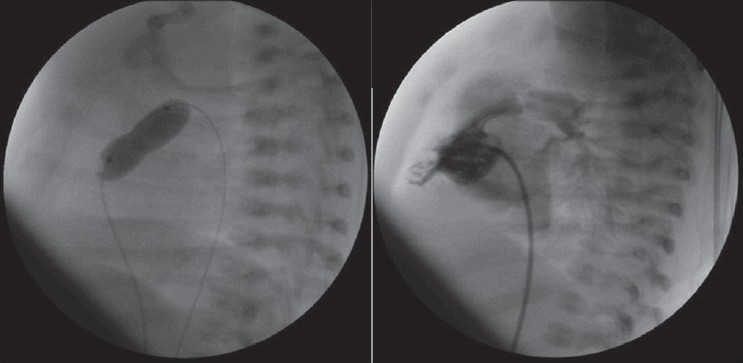
Perforation of atretic pulmonary valve using a guide-wire and then pulmonary balloon valvuloplasty after ductal stenting

Ductal stent implantation was performed in four cases for augmentation of pulmonary blood pressure and to increase the patient’s oxygenation [Table 2, Figure 6]. In two patients with critical pulmonary stenosis, the stent implantation alone was enough for an optimal result. But, in a patient with pulmonary atresia with intact ventricular septum, oxygen saturation did not increase efficiently after stenting of ductus. The patient had pulmonary atresia of membranous type and a well-developed right ventricle, and did not have any right ventricle-dependant coronary circulation. In a retrograde fashion, atretic pulmonary valve was perforated with a guide-wire and then pulmonary balloon valvuloplasty was performed with resultant antegrade pulmonary flow [Figure 7].

In other case who had double inlet left ventricle, double outlet left ventricle, left modified BT shunt (obstructed), PDA (small), in addition to palliative pulmonary balloon valvuloplasty, ductal stenting was performed due to oxygen saturation did not increase efficiently after the first procedure [Table 2].

ASD closure using Amplatzer or Cardi-O-Fix atrial septal occluder was performed with ductal coil embolization in two patients, and with pulmonary balloon valvuloplasty in two patients [Table 2]. The sizes of ASD’s were moderate, and Qp/Qs calculated at cath-lab were more than 1.5 in all cases. They found no residual shunt on anjiocardiogram in any patient after procedure.

Balloon atrial septostomy was performed successfully in one, coil embolization of pulmonary sequestration in one, stent implantation to a long-segment coarctation in one, and recanalization and balloon angioplasty to a surgically created aortopulmonary shunt in two patient [Table 2].

The mean procedural time was 97.9 ± 43.4 minutes (58-240 minutes) and the mean fluoroscopy time was 27.6 ± 10.9 minutes (11–47 minutes).

There was no mortality related to any combined procedure.

### Patient symptoms

Most of the patients who underwent the combined transcatheter procedure were asymptomatic. Six patients (under 3 months of age) with aortic coarctation who had balloon angioplasty done all had pulmonary hypertension, and two patients had left ventricular systolic dysfunction. Respiratory distress was present in all patients. Two-day-old patient with Shone’s Complex presented with slight confusion, paleness and acrocyanosis. Another 6.5-year-old patient with a wide secundum ASD and narrow PDA had stage 2 dyspnea according to NYHA criteria. After balloon angioplasty was performed on five patients with aortic coarctation, their symptoms subsided completely. In the patient with Shone’s Complex the tachypnea and cardiac deficiency were only partially alleviated. Patients with prominent cyanosis and critical pulmonary stenosis or cyanotic congenital heart disease saw considerable decrease in cyanosis after the procedure.

### Follow-up

The mean follow-up time and the mean number of follow-up were 22.4 ± 24.7 months (1 days-72 months) and 5.6 ± 3.8 times (once-13 times), respectively.

Recoarctation was developed in four out of 12 coarctation patients (33.3%). Diagnoses, ages of combined procedure and redo balloon angioplasty, and outcomes of redo balloon angioplasty of these cases have been shown in Table 3. In one of them, 6 months after redo balloon angioplasty of aortic coarctation, recoarctation was developed again, and this patient was sent to surgery for resection and end-to-end anastomosis.

**Table 3:** Patients underwent redo balloon angioplasty for aortic coarctation and outcomes of the redo intervention

Cases	Diagnosis	Age of combined procedure	First part of combined procedure	Second part of combined procedure	Age of redo intervention	Outcomes
1 ANB	AoCo, Bicuspid AV, AS, LVH, PFO	1 month	ABV	BAP	7 months	AoCo gradient decreased from 30 to 16 mmHg.
2 SEB	AoCo, bicuspid AV, AS, LVH, PDA, PFO, severe PH	7 days	ABV	BAP	16 months	AoCo gradient decreased from 32 to 8 mmHg.
3 NAO	Shone complex, critic AoCo, mitral stenosis (mild), bicuspid AV, AS, Pm VSD, PDA, left ventricular hypoplasia (mild), systolic dysfunction	2 days	BAP	ABV	3 weeks	Systolic dysfunction improved highly, but pulmonary hypertension did not decrease.
4 YEC	AoCo, Pm VSD, left ventricle-right atrium communication, left ventricular systolic dysfunction, PFO, pulmonary sequestration	1 month	BAP	Coil embolization of the pulmonary sequestration	3.5 months	AoCo gradient decreased from 56 to 5 mmHg.

AoCo, Aortic coarctation; AV, Aortic valve; AS, Aortic stenosis; ABV, Aortic balloon valvuloplasty; BAP, Balloon angioplasty of aortic coarctation; LVH, Left ventricular hypertrophy; PDA, Patent ductus arteriosus; PFO, Patent foramen ovale; PH, Pulmonary hypertension; Pm, perimembranous; VSD, Ventricular septal defect.

In an infant with Shone’s complex, systolic dysfunctions and pulmonary hypertension were improved partially in the first week of combined procedure including coarctation angioplasty and aortic balloon valvuloplasty. But this patient had mild left ventricular hypoplasia and repeated echocardiographic examinations revealed tunnel type stenosis at left ventricular outflow tract in addition to aortic valvular stenosis. At follow-up, severity of subaortic stenosis increased. Balloon angioplasty for aortic coarctation was performed again at 3-weeks-old of his life due to persisted systolic dysfunction and pulmonary hypertension. After second balloon angioplasty, systolic function improved highly (ejection fraction increased from 39 to 52%), but pulmonary hypertension did not decrease. Surgery was planned for subaortic stenosis, because of a maximum gradient of 96 mmHg (mean 63 mmHg) measured at last examination, but this patient died before surgery.

In the cases underwent balloon atrial septostomy and balloon angioplasty for aortic coarctation, arterial switch, VSD closure and coarctation repair-resection and end-to-end anastomosis were performed at 1-month age. Because of neopulmonary valve stenosis developed, this patient was operated again at 4 years of age.

We considered magnetic resonance imaging of arcus and descending aorta, although we did not detect and aneurism formation after coarctation angioplasty in any patient.

Pulmonary restenosis did not occur in follow-up of any patient who underwent pulmonary balloon valvuloplasty except palliative pulmonary valvuloplasty as a first or second procedure. Two patients with Fallot’s tetralogy underwent palliative pulmonary balloon valvuloplasty was totally corrected after a non-eventful follow-up period.

In one of the two patients with critic pulmonary stenosis underwent ductal stenting, it is detected that the flow of ductal stent occluded at follow-up. The oxygen saturation of this case was good, and there was no gradient through right ventricular outflow tract. In the other case with critic pulmonary stenosis, tricuspid valve was hypoplastic and right ventricle was hypoplastic and bipartiete. During follow-up, while pulmonary restenosis has not been observed and the flow of ductal stent has been persisted, right ventricle and tricuspid valve has not been improved, and univentricular repair was planned for this patient. For this end, cardiac catheterization and angiography was performed 15 months after her combined procedure and her pulmonary index was found enough for Glenn’s anastomosis.

At the same time, in the patient with double inlet left ventricle, double outlet left ventricle, pulmonary stenosis and left modified Blalock-Taussig shunt (obstructed), the flow of ductal stent and oxygen saturation did not decrease until now. Elective univentricular repair was planned for this patient too.

Balloon dilatation of ductal stent with a 4.5 mm coronary balloon was performed in a patient who has pulmonary atresia with intact ventricular septum and patent ductus arteriosus 1 month after his combined procedure including ductal stent implantation and perforation of atretic valve with subsequent pulmonary valvuloplasty, because of a narrowing in-stent diameter and decrease in oxygen saturation. After dilatation of ductal stent, oxygen saturation increased and stayed in normal levels at follow-up.

Restenosis was not observed in patients who underwent aortic balloon valvuloplasty as a part of combined procedure during follow-up period except when a patient died.

In one patient who has not suffered from aortic insufficiency after combined procedure, minor aortic regurgitation developed after about 2 years. Other than this, in another patient mild aortic insufficiency developed after combined procedure, aortic regurgitation increased from mild-to-moderate 18 months after aortic balloon valvuloplasty. In two patients developed mild aortic insufficiency after combined procedure, aortic insufficiency has been persisted as mild grade. The aortic regurgitation reduced to mild at follow-up, in a patient with moderate aortic regurgitation detected just after the combined procedure.

Residual ductal shunt disappeared in 3 months in a case that a tiny residual defect was detected after embolization procedure. We did not detect any coil migration or stenosis at left pulmonary artery.

A temporary pacemaker was positioned by intravenous route and dexamethazone was started to the patient who developed complete atrioventricular block after VSD closure. The patient recovered in 3 days. The case in which VSD was closed by non-detachable coil, the severity of the aortic regurgitation was reduced from mild-to-trace level in follow-up.

## DISCUSSION

With recent advances in interventional catheterization, single or combined transcatheter interventions made the surgical treatment easy in some multiple congenital cardiac diseases, and even disappeared the need for surgery in some.[1]

Today, pulmonary balloon valvuloplasty has become the first choice in nearly all of the valvuler pulmonary stenosis. Likewise, coarctation angioplasty and stenting procedures has became the procedure of choice in native and postoperative coarctation in selected cases.[6–8] Although aortic balloon valvuloplasty has been performed for palliation mostly, it may have provided the cure in some cases. In many centers in all around the world, transcatheter PDA closure, VSD, ASD, fistule and collateral closure in selected cases, stent implantation to ductus, pulmonary artery and its branches have been carried through with success.[9–14]

In cases with multiple congenital cardiac defects, different transcatheter interventions may be performed in several different sessions, or may be performed in the same session as a combined procedure.[15,16] The first case of combined transcatheter intervention reported in the literature was a 7-year-old girl whose ASD was closed by an Amplatzer Septal occluder with pulmonary balloon valvuloplasty. Recently, reports on combined interventions have been increased tremendously.[17–19]

Introduction of combined transcatheter procedures lessened the ratio of surgery needed cardiac lesions and decreased the mortality and morbidity related to surgery. In addition to this, combined procedures have led the surgery to be delayed to more elective conditions. In our series, at the follow-up of four cases that underwent aortic balloon valvuloplasty or coarctation angioplasty at before 6 months of age, there was no need to repeat the procedure.

Today, surgery is recommended in neonatal and early infantile native aortic coarctation due to increased risk of recoarctation after balloon angioplasty. But if there is any accompanying lesion or systolic dysfunction, the combined transcatheter procedures may be the procedure of choice.[20] A 1-month-old baby with LV systolic dysfunction underwent coarctation angioplasty with embolization of pulmonary sequestration in the same session. Recoarctation developed at the age of 3 and 5 months and coarctation angioplasty was repeated. At the age of 9.5 months, the patient had to be sent for surgery by recurrence of recoarctation.

Combined interventional procedures may totally obviate the need for surgery in some of the patients. We successfully closed five VSDs that fulfill the criteria for surgical closure. VSDs were closed by using different devices, and so obviated the need for surgery in these patients. In one of the patients, the indication for VSD closure was aortic regurgitation. In another patient, the indication was aortic valve prolapse and aortic regurgitation. The aortic regurgitation decreased from mild to trivial amount after procedure in both cases.

In another patient with long-segment coarctation, successful stent implantation to diseased segment obviated the need for surgery and maintained the total cure for coarctation.

Today, in selected cases ASDs and VSDs can be successfully closed with different devices.[21–26] Some authors reported ASD and VSD closures as the former procedure of a combined intervention, with an embolization of a PDA or a pulmonary balloon valvuloplasty performed after those procedures.[18,27,28] In our study, coil embolization of ductus in four and coarctation balloon angioplasty in one patient were successfully performed as second procedures of combined interventions after percutanous VSD closure as the first procedure. The successful ASD closure was performed in the same session with ductus embolization in two, and with pulmonary balloon valvuloplasty in two patients.

Combined transcatheter interventions may sometimes obviate the need for some of the stages of a complex surgical procedure, in selected cases. For example, transcatheter pulmonary valvulotomy and valvuloplasty can be a treatment of choice for neonates with pulmonary atresia with intact ventricular septum, if there is a patent infundibulum, no right-ventricular-dependent coronary circulation, and adequate tricuspid valve and pulmonary valve.[29] In our series, a combined procedure of ductal-stent implantation with retrograde perforation of atretic pulmonary valve and subsequent balloon valvuloplasty was performed in a patient with pulmonary atresia with intact ventricular septum with no RV-dependent coronary circulation and well- developed RV. On the other hand, in some patients planned univentricular repair, ductal stenting or palliative pulmonary balloon valvuloplasty can obviate the need for modified Blalock-Taussig shunt. In our two cases planned univentricular repair, we have not needed performing the modified Blalock-Taussig shunt until now.

The advantages of the interventional treatment compared to the surgical approach are obvious. Although mortality is rarely seen after surgery, morbidity related to thoracotomy and pericardiotomy, cardiopulmonary bypass and possible risks of blood transfusions are more frequently observed.[30] But it must be kept in mind that increased procedural time and fluoroscopy time in combined procedures may be disadvantageous. Although the follow-up time is not long enough, we did not detect any complication in our cases.

All possible combinations can be considered for combined transcatheter procedures. But it is problematic to decide which procedure must be the followed first. Song *et al*. suggested that the difficult one must be the first then the easy, the complex one first and then the simple, and latter manipulations should not diminish the therapeutic effects of the former procedure.[18] For example, some authors advice to perform coarctation angioplasty first and then aortic balloon valvuloplasty, in patients having both valvular aortic stenosis and aortic coarctation. Contrarily some other authors argued that this is only a technical choice, and they did not report any serious hemodynamic problem in cases that they perform aortic valvuloplasty first.[16]

We performed aortic balloon valvuloplasty first in five patients, and coarctation angioplasty first in one patient. In a two-day-old patient in a bad condition with LV systolic dysfunction, we performed the coarctation angioplasty first, because we thought the coarctation had the more deleterious effect on the patient’s condition. In our series, we mostly choose the hemodynamically more important and harder procedure as the first intervention.

In conclusion, for the treatment of combined congenital cardiovascular defects, multiple transcatheter interventions in the same session are feasible, safe and effective with satisfactory good results. Second intervention may be performed as complementary procedure or independently to the first intervention.
